# The Effects of Food on Cannabidiol Bioaccessibility

**DOI:** 10.3390/molecules26123573

**Published:** 2021-06-11

**Authors:** Khadijeh Mozaffari, Stephanie Willette, Ben F. Lucker, Sarah E. Kovar, Francisco Omar Holguin, Ivette Guzman

**Affiliations:** 1 Department of Plant and Environmental Sciences, New Mexico State University, Las Cruces, NM 88003, USA; mozafari@nmsu.edu (K.M.); frholgui@nmsu.edu (F.O.H.); 2 Trait Biosciences, Los Alamos, NM 87544, USA; stphnwlltt@comcast.net (S.W.); bflucker@traitbio.com (B.F.L.); kovar.sarah.e@gmail.com (S.E.K.)

**Keywords:** CBD, hemp, cannabis, in vitro digestion, fasted digestion, fed digestion, UPLC-MS, metabolites

## Abstract

Cannabidiol (CBD) is a hydrophobic non-psychoactive compound with therapeutic characteristics. Animal and human studies have shown its poor oral bioavailability in vivo, and the impact of consuming lipid-soluble CBD with and without food on gut bioaccessibility has not been explored. The purpose of this research was to study the bioaccessibility of CBD after a three-phase upper digestion experiment with and without food, and to test lipase activity with different substrate concentrations. Our results showed that lipase enzyme activity and fatty acid absorption increased in the presence of bile salts, which may also contribute to an increase in CBD bioaccessibility. The food matrix used was a mixture of olive oil and baby food. Overall, the fed-state digestion revealed significantly higher micellarization efficiency for CBD (14.15 ± 0.6% for 10 mg and 22.67 ± 2.1% for 100 mg CBD ingested) than the fasted state digestion of CBD (0.65 ± 0.7% for 10 mg and 0.14 ± 0.1% for 100 mg CBD ingested). The increase in bioaccessibility of CBD with food could be explained by the fact that micelle formation from hydrolyzed lipids aid in bioaccessibility of hydrophobic molecules. In conclusion, the bioaccessibility of CBD depends on the food matrix and the presence of lipase and bile salts.

## 1. Introduction

*Cannabis sativa* contains a diverse set of phytocompounds such as cannabinoids, terpenes, carbohydrates, fatty acids, and phenolic compounds [1]. Notably, cannabinoids are the most sought after phytocompounds in *C. sativa* and are synthesized in the trichomes of the plant [1]. Among the different *C. sativa* chemotypes that have been identified, those high in Δ9-tetrahydrocannabinol (THC) are commonly called marijuana and used for both medicinal and recreational purposes. The other chemotypes that are non-psychoactive, rich in fiber and cannabidiol (CBD) while low in THC, are known as hemp or industrial hemp. These chemotypes are used for food, medicine, and textile production [2,3]. The most predominant cannabinoids present in industrial hemp are cannabidiolic acid (CBDA) and cannabigerol acid (CBGA), precursors to CBD and cannabigerol (CBG), respectively [3].

CBD consumption, either as pure edible extract or inhaled, has been reported to reduce tobacco and cigarette use, to have cardiac and extracerebral benefits, promote calmness, aid in sleep, work as an antiepileptic through reduction in seizures, have anti-inflammatory, antimicrobial, and antioxidant properties, and to act as a neuroprotectant [4,5,6,7,8,9,10,11,12,13]. Cannabinoids interact with two G-protein-linked cannabinoid receptors (CB_1_ and CB_2_) [13]. The CB_1_ receptor is in the brain and also localized in the central nervous system while the CB_2_ receptor is highly expressed in peripheral organs with immune function and hence is upregulated in response to immune cell activation and inflammation [14]. Many of the psychoactive analgesic effects of THC are due to its interaction with the CB_1_ receptor, while the non-psychoactive cannabinoids, like CBD, have a lower affinity for both CB_1_ and CB_2_ receptors [3].

Previous studies have focused on the oral and intraperitoneal pharmacokinetics of THC and CBD together in human plasma and also on CBD in humans, dogs, rats and pigs [15,16,17,18,19]. However, despite many publications on cannabinoid metabolism and distribution in human and animal studies, there is a lack of information regarding the absorption and bioavailability of cannabinoids. Bioavailability includes digestion, absorption, metabolism, distribution, and bioactivity of the ingested compound. In general, the fraction of the total ingested compound that reaches systemic circulation is defined as bioavailability [20]. Bioaccessibility is defined as the quantity of a compound that is released from the food matrix and becomes available for absorption by the intestine epithelial cells [20]. While the information on CBD bioaccessibility in humans is incomplete, animal studies on rats, mice, and piglets for CBD oral and absolute bioavailability show low bioavailability [21,22]. For example, in a study where Rhesus monkeys received an oral dose, CBD was only 16% bioavailable on average [23]. In addition, the 16% bioavailability was lower than that in another study where CBD solution was administered orally to rats [21]. Also, bioavailability following oral administration of CBD to dogs was low due to the first pass effect [24]. Extremely poor oral bioavailability of CBD in in vivo studies might be due to its poor water solubility, or metabolism to other metabolites such as 7-OH CBD via CYP3A4 and CYP2C19 enzymes, or glucuronidation, resulting in clearance [25,26]. Therefore, understanding CBD bioaccessibility and absorption following oral administration is very important to comprehend the challenges in dose dependent administration and provide insight for formulation and drug delivery to increase oral bioavailability of CBD and other cannabinoid species in humans.

The uptake of ingested plant-based bioactive compounds by the human digestive system takes place in the small intestine and is achieved by a diverse group or transporters, carrier proteins, hydrolases in epithelial cells and is affected by many factors [27,28,29]. One factor is compound polarity considering there are different absorption mechanisms for hydrophobic and hydrophilic compounds [27]. Another factor is the non-covalent interactions of health promoting plant-derived compounds such as flavonoids with macronutrients in the food matrix [27,29]. Moreover, the role of lipids on metabolite bioaccessibility has previously been published, showing that fatty acids are hydrolyzed from food triacylglycerides by lipases, and form micelles in the presence of bile salts [27,30,31,32,33,34,35,36]. The micelle formations are important for capturing hydrophobic food compounds and allowing for uptake by epithelial cells in the gut. In one study, the addition of olive oil to carrot samples during the cooking process increased micellarized carotene after an in vitro digestion [30]. In another study, the intestinal bioavailability of carotenoids was enhanced using olive oil and soybean oil, since unsaturated fatty acids present in these oils act as suitable carriers for carotenoids [31,37]. Similar observations have been reported in lipid-soluble cannabinoids, and the co-administration of lipids and cannabinoids increased the bioavailability of CBD and THC in rats by 2.5-fold and 3-fold, respectively [32]. Therefore, lipase activity is important to validate when performing simulated human digestion of foods where hydrophobic compounds are mixed with oils and food.

To further understand CBD bioavailability in humans, an in vitro digestion model was optimized and employed to study the bioaccessibility of naturally occurring lipid-soluble CBD digested with and without food. For the three-phase in vitro digestion system, experiments were conducted to validate the activity of two enzymes involved in the oral and gastric phase. In addition, an assay was designed to test our hypothesis that the lipase enzyme would cleave fatty acids from triacylglycerides, thus promoting micellarization of fat-soluble compounds like CBD, in the presence of bile salts and absence of colipase enzyme. Additionally, to measure the bioaccessibility of lipid-soluble CBD, digestions were performed for the compound with and without food. We hypothesized that CBD would be more bioaccessible due to higher micellarization efficiency in fed state digestion compared to fasted state digestion.

## 2. Materials and Methods

### 2.1. Reagents and Chemicals

Hemp extracted CBD isolate was kindly provided by Trait Bioscience Inc., Los Alamos, NM, USA. Acetonitrile, water, and methanol were all ultra-performance liquid chromatography—mass spectrometry (UPLC-MS) grade and purchased from VWR (Radnor, PA, USA). Formic acid was obtained from Sigma-Aldrich (St. Louis, MO, USA). All digestive enzymes (α-amylase, β-glucosidase, pepsin, pancreatin, lipase, and bile salts) were obtained from Sigma-Aldrich (Milwaukee, WI, USA).

### 2.2. Gastric and Intestinal Phase Lipase Activity

The lipase, pancreatin and bile were purchased from Sigma-Aldrich. The p-nitrophenyl palmitate (pNPP), a water-soluble compound (N2752, Sigma-Aldrich), was used as a substrate for the lipase enzyme as previously described [34]. Upon hydrolysis of palmitate from pNPP, the resulting product is 4-nitrophenol (4-NP), and can be measured spectrophotometrically at 410 nm. Quantification of the product after the assay was achieved with a standard curve of 4-NP (1048, Sigma-Aldrich) dilutions in phosphate buffer.

Final assay enzyme concentrations were 1:2 mg/mL lipase:pancreatin and 1:2:3 mg/mL lipase: pancreatin:bile using 96 well plates separately just as in the artificial digestion protocol. Controls consisted of phosphate buffer and lipase enzyme without substrate pNPP. Experimental wells contained substrate pNPP in phosphate buffer and the lipase enzyme. All experimental and control wells were performed in triplicate. Temperature was set at 37 °C and the spectrophotometer was set to collect kinetic data over 2 h for the lipase: pancreatin (1:2) experiment. Substrate concentrations were 0.1 mM and 1.0 mM and were made from the 10.0 mM stock solution of pNPP in isopropanol. 4-NP dilutions in phosphate buffer were used to make a standard curve ranging from 5–1000 µM.

On a separate 96 well plate, the same procedure was followed for the lipase: pancreatin: bile salt (1:2:3) experiment. Control and experimental samples were the same as mentioned above for the 1:2 mg/mL lipase: pancreatin experiment. However, interference was observed in sample wells containing bile salts; therefore, the enzymatic reaction time was reduced to 30 min to prevent interference and detector saturation. After an incubation period of 30 min at 37 °C, 25 µL of the reaction mixture (supernatant) was taken and immediately mixed with 1 mL of 0.1 N NaOH in a 1 mL cuvette to stop the reaction and dilute the sample. The liberated pNP was detected at 410 nm, and bile salt interference was corrected by using a blank without substrate for background subtraction. The reaction rates for this experiment were obtained from the slope of the absorbance versus the time curve by using an extinction coefficient for the p-nitrophenol (pNP) that was determined from the absorbance of pNP as a standard solution.

### 2.3. Fasted State In Vitro Digestion of CBD

The three-step in vitro digestion employed to mimic the human digestive tract in this study consisted of an oral phase, a gastric phase, and an intestinal phase. The method used was modified slightly from a previously described protocol [38]. For the fasted state digestion oral phase, 10 mg and 100 mg of CBD were added to 1 mL distilled water in a 50 mL pyrex glass centrifuge tube (DAIGGER Scientific, Hamilton, NJ, USA) and mixed with 1 mL oral fluid. The mixtures were vortexed for 30 s and immediately the gastric phase of digestion was started by adding pepsin into NaHCO_3_ and the pH was adjusted to 2.5. The mixtures were blanketed with nitrogen gas, wrapped in parafilm, and shaken in a 37 °C incubator for 1 h in the dark at 95 rpm. Meanwhile, the intestinal phase was prepared (Table S1) by mixing bile in NaHCO_3_ and sonicating it for 45 min. For the intestinal phase, the pancreatin-lipase (2 mL of 2:1 ratio) was mixed into the same solution as bile salt. The enzyme solution and the bile solution were added to the tube containing the gastric phase and the pH was adjusted to 6.5 using 1N NaOH. The digestion sample tubes were blanketed with nitrogen and shaken for another 2 h at 37 °C in the dark at 95 rpm. After completion of the intestinal phase, 4 mL of the resulting Digesta (DG) was transferred into conical tubes, diluted with acetonitrile DG: Acetonitrile 1:2 (*v*/*v*), blanketed with nitrogen, and kept at −80 °C for analysis by UPLC-MS. The remaining volumes of the DG samples were centrifuged at 10,000× *g* at 4 °C for 60 min (Beckman L7-65 Ultracentrifuge, Beckman Instruments, Palo Alto, CA, USA). After centrifugation, the aqueous supernatant (AQ) containing the micellar fractions were filtered through the 0.22 µm pore (VWR, USA), diluted with acetonitrile AQ: Acetonitrile 1:2 (*v*/*v*) and stored at −80 °C for further analysis. The insoluble pellet was dissolved into the acetonitrile 1:2 (*v*/*v*) for further analysis.

### 2.4. Fed State In Vitro Digestion of CBD

For the fed in vitro digestion experiment, CBD was mixed with foods and oil. Briefly, 10 mg and 100 mg CBD were each first mixed with 0.5 g olive oil, and then added to 2 g baby food (Turkey and Gravy, Gerber, IL, USA). The oral phase samples were vortexed for 30 s followed by the gastric and intestinal phase protocols mentioned above. After the completion of the three-phase in vitro digestion procedure, 15 mL of the DG sample was collected, blanketed with nitrogen and kept at −80 °C for further analysis. The rest of the DG sample was centrifuged at 10,000× *g* for 1 h at 4 °C and the AQ fraction was collected, filtered, diluted in acetonitrile 1:2 (*v*/*v*) and blanketed with nitrogen and kept at −80 °C for the UPLC-MS analysis.

For the creation of CBD calibration curves, a series of eight dilutions were prepared for CBD standard in methanol. Their concentrations ranged from 3–150 μg/mL, and they were done in triplicates.

### 2.5. UPLC-MS Chromatograms of CBD Standard

In order to automatically detect and quantify CBD in the pre- and post-digestion fractions using UPLC-MS analysis, standard curves for CBD were prepared in methanol at 1 mg/mL concentration. In Figure S1, the extracted ion chromatograms are shown with the corresponding mass spectrum for CBD standard.

### 2.6. Digestion Extractions of CBD and UPLC-MS Analysis

Diluted AQ and DG fractions were filtered using the 0.2 µm nylon filters for DG fraction and injected for analysis by ultra-performance liquid chromatography-mass spectrometry (UPLC-MS) to obtain CBD concentration values. Percent (%) recovery values were calculated for CBD using the following equation:

(1) $$\%\;Recovery=\frac{Concentration\;in\;AQ}{Concentration\;in\;DG}\times 100$$

All solvents (acetonitrile, water, methanol, formic acid) were UPLC-MS grade and purchased from VWR (Sparks, NV, USA). The MS instrument employed was a Waters QTOF/MS (Waters Corp., Milford, MA, USA). Ionization was performed in both positive and negative ion modes. The sample injection volume was 5 uL and the elution gradient was 5–95% acetonitrile in 0.1% formic acid over a 25 min period. The UPLC-MS column used was a C-18 reversed-phased column (150A C18 column, 20 mm length, 2.1 mm internal diameter from Millipore) using a mobile phase of water in 0.1% formic acid (*v*/*v*) (solvent A) and acetonitrile in 0.1% formic acid (*v*/*v*) (solvent B). Elution was performed at a flow rate of 0.25 mL/min. The MS source temperature was set at 110 °C, and the desolvation temperature was set at 300 °C with desolvation gas flow at 500 L h^−1^. The capillary voltage was 3 kV. The mass spectra were recorded across the range of *m*/*z* 100 to 1500 Da. Masslynx V4.1 was used to automatically quantify the compounds based on the calibration curve of the standard.

CBD was quantified in pellet (P), digesta (DG) and aqueous (AQ) fractions by liquid chromatography—mass spectrometry. Digestive stability, the percentage recovery of CBD in DG fraction was calculated by dividing the moles of CBD quantified in DG by the moles of CBD quantified in raw material (RM) multiplied by 100. The micellarization efficiency is the percentage of CBD recovered in the AQ phase of digestion and was calculated by dividing the moles of CBD in AQ by the moles of CBD in DG multiplied by 100.

### 2.7. MS/MS Survey of Metabolites

To further confirm the presence of any metabolites in the DG, AQ, and P phases, an MS/MS survey was performed on negative ion mode with the inclusion list of all the masses for the expected metabolites (Table S2). The setting for TOF MSMS function was as follows: centroid data format, negative mode and polarity, mass rages from 100 to 1000, time 0 min to 25.0 min and scan time 2.1 s with inter scan time of 0.1 s. The instrument parameters were as follows: capillary 3000.0 V, sample cone 35.0 V, source temperature 100 °C and collision energy 5.0.

## 3. Results

### 3.1. Gastric and Intestinal Phase Lipase Activity

**Figure 1:**
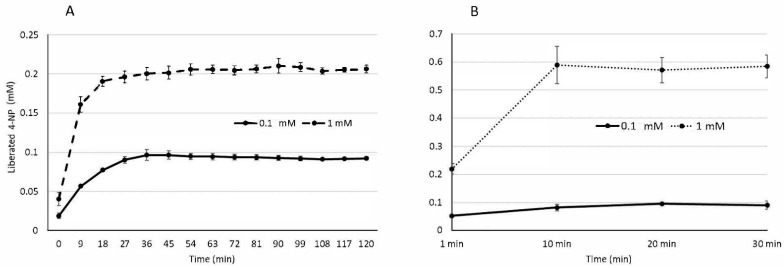
Enzyme kinetics graph of liberated reaction product, NP, over time from two substrate concentrations (1 mM and 0.1 mM NPP) and two enzyme and bile combinations (**A**) lipase:pancreatin (1:2) (**B**) lipase:pancreatin:bile (1:2:3). Values are averaged from three biological replicates (*n* = 3). ±SE bars are displayed.

To assess the effects of lipase and pancreatin enzymes on lipolysis, we evaluated the activity of lipase and pancreatin (at the same concentration used in in vitro digestion) in the presence and absence of bile salt. Free fatty acids are required for hydrophobic compounds to be taken up by human epithelial cells through micellarization. The digestion and absorption of fats is a complex process that is affected by several different factors. One crucial factor is the solubilization of lipids in the gut that occurs in the presence of bile salts. Bile salts are adsorbed onto the lipids’ surfaces to aid in the hydrolysis and release of free fatty acids through complex formation with lipase and pancreatin. Subsequently, free fatty acids are incorporated within mixed micelles containing bile salts allowing for absorption by intestinal brush borders [39,40]. An important role of bile salts has also previously been shown in fat digestion and lipolysis [41,42,43]. Therefore, the process of mixing bile salts and lipids to form micelles aids in lipid solubilization [44]. Two enzyme combinations were tested to measure enzyme activity using the substrate p-nitrophenyl palmitate (pNPP) at two concentrations (0.1 mM and 1 mM). The reaction product, 4-nitrophenyl (4-NP), was detected spectrophotometrically at 410 nm and quantified using a 4-NP standard curve (Figure 1).

The hydrolysis of pNPP increased with substrate concentration (Figure 1A). Moreover, when increasing the substrate amount from 0.1 mM pNPP to 1.0 mM pNPP, 4-NP production increased from 20.6 to 58.6% (Table 1). Additionally, bile salts increased lipase activity when added to lipase and pancreatin. However, there was no statistically significant increase between experiments with and without bile (Figure 1B, Table 1 and Table S3). Our results showed an increase in 4-NP liberation as a product over time using the lipase and pancreatin mixture in the presence of bile salt with a substrate concentration of 1.0 mM pNPP (Figure 1). The increase of 4-NP in the presence of bile salts could also be due to an increase in the solubilization of pNPP, i.e., an increase in the initial substrate concentration available to the enzyme. Another explanation may be that bile salts stimulate lipase activity and therefore, could enhance lipolysis in digestion thus improving fatty acid absorption and micelle formation. More importantly, it showed no inhibition of lipase activity of the pancreatin/lipase complex in the absence of an independent colipase.

### 3.2. Fasted State In Vitro Digestion of CBD

#### 3.2.1. Percent (%) Recovery

**Figure 2:**
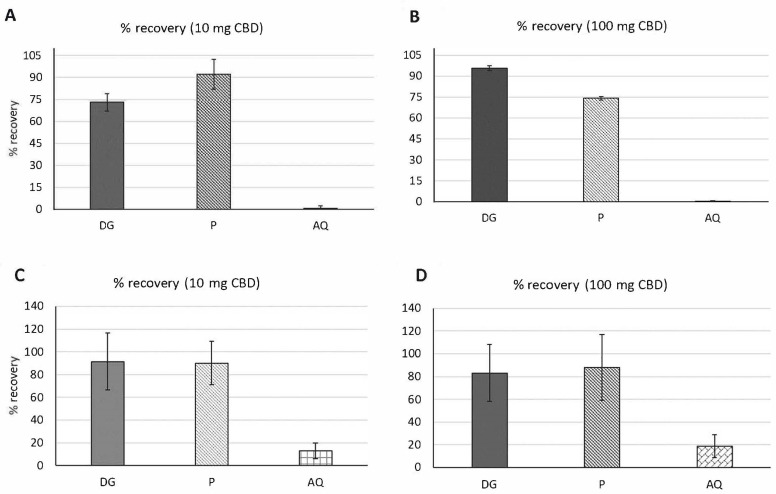
Percent (%) recovery of CBD after fasted state (**A**,**B**) and fed state (**C**,**D**) digestions. (**A**): % recovery using 10 mg CBD, (**B**): 100 mg CBD in DG, P and AQ fractions after complete fasted state digestions. (**C**): % recovery using 10 mg CBD, (**D**): 100 mg CBD in DG, P and AQ fractions after fed state digestions. DG: Digesta, AQ: Aqueous and P: Pellet. Values are means of ±SE. (*n* = 3).

For the fasting digestion, or a digestion without any food, % recovery of the CBD was measured after the digestion in the digesta (DG), aqueous (AQ), and pellet (P) samples. When digesting 10 mg CBD, a higher percentage of CBD was recovered in the P fractions (92.22 ± 10%), while the 100 mg CBD digestion showed a higher % recovery in the DG samples (95.67 ± 2%) (Figure 2A,B and Table 2). The aqueous (AQ) sample was the supernatant collected by centrifuging the entire digestion (digesta, DG) and contains the fraction of metabolites and micelles that are accessible for absorption by epithelial cells. For both 10 mg and 100 mg CBD digestions, the AQ micellar fraction contained the lowest amount of CBD. Moreover, there were no significant differences in the recovery of CBD from the AQ fractions of the 10 mg and 100 mg CBD digestions. Low bioavailability in the fasted state digestion experiment was expected due to the absence of fats to promote micellarization of CBD. We observed significantly higher CBD recovery in the DG fraction compared to the AQ fraction for both 10 mg and 100 mg CBD digestions.

#### 3.2.2. Bioaccessibility

Micellization efficiency is defined as the percentage of CBD recovered in the AQ fraction, and digestive stability as the percentage of CBD recovered in DG. For the fasted state digestions of CBD, the results are shown in Table 2. The digestive stability was 72.99 ± 6% for 10 mg CBD and 95.67 ± 2% for the 100 mg CBD (Table 2). The micellar efficiencies for CBD in the fasted state digestions were very low (0.65 ± 0.7% for 10 mg CBD and 0.14 ± 0.1% for 100 mg CBD), and were not statistically significant from each other (Table 2).

### 3.3. Fed State In Vitro Digestion of CBD

#### 3.3.1. Percent (%) Recovery

Many athletes and patients prefer oral administration of CBD or other cannabis-based products [45]. Consumption of cannabis (not specifically CBD) with cookies, lipids or other foods was previously reported to improve their formulations [32]. A recent study tested the oral bioavailability of cannabinoids such as THC and CBD with sesame oil in rats and concluded that 30% of the THC and CBD was bioavailable in the micellar fractions [32]. However, there has been no previously published studies on the bioavailability of CBD with food. For the fed digestion experiment, CBD was added to 2.5 g baby food and olive oil (4:1 ration baby food to olive oil). The percent recoveries of CBD after the fed digestion experiments are shown in Figure 2C,D and Table 2 for DG, P, and AQ samples. After digesting 10 mg CBD, a significantly higher recovery was obtained in the DG and P fractions relative to the AQ fraction, which were 91.39 ± 25% and 90.15 ± 18%, respectively (Figure 2C). There were no significant differences between the recovery percentages of CBD from the 10 mg CBD in the fed digestion AQ compared to 10 mg CBD in the fasted digestion AQ. Similarly, there was no statistically significant increase in CBD recovery in the AQ fraction from the 100 mg CBD fed digestion versus the 100 mg CBD fasted digestion (Figure 2 and Table S4). Moreover, we observed higher % recovery of CBD from the AQ fraction after digesting 100 mg CBD (18.59 ± 10%) compared to the digestion of 10 mg CBD (12.93 ± 7%) but it was not significant (Figure 2D, Table 2 and Table S4). This implies that the bioaccessibility of CBD can be increased in the presence of fatty foods.

#### 3.3.2. Bioaccessibility

The digestive stability and the micellerization efficiency of CBD digested with baby food and olive oil are displayed in Table 2.

The effects of a medium-high fat, medium calorie meal on the bioavailability of CBD was assessed for two doses (10 mg, and 100 mg). The bioaccessibility of CBD was tested using a mixture of olive oil and baby food as a food matrix. A significantly higher micellar efficiency was measured for CBD in the AQ fraction of the fed state (18.59 ± 10%) compared to the fasted state (0.15 ± 1%) after digesting 100 mg CBD (Figure 2). Due to the presence of lipids in the olive oil and baby food during the digestion and CBD’s highly lipophilic property, we did find a higher percentage of CBD in the AQ micellar fraction, proving our hypothesis. In the current study, the use of bile salts along with the olive oil and baby food recovered about 22.6% of CBD in the micellar fraction. We observed a 22-fold increase in CBD recovery in the micellar fraction in the fed digestion compared to the fasted state digestion. Moreover, human studies on oral bioavailability of CBD in vivo using different CBD formulations showed greater CBD bioavailability in fed patients [19]. However, the difference in the micellarization efficiency between starved digestion and fed digestion may not reflect the same extent of difference in oral bioavailability in vivo. Another study reported 30% CBD recovery in the micellar fraction that is the amount bioavailable for absorption using sesame oil [32]. This difference might be due to their lipid-based formulation of CBD which likely played an important role in solubility and bioavailability of CBD. Another reason might be the presence of proteins in the baby food we used that interact with CBD and reduce CBD bioavailability. Researchers have also shown the negative impact of non-covalent interactions between proteins and flavonoids such as lipid soluble quercetin, which led to aggregation and solubility loss [28]. Significantly higher micellarization efficiency was obtained from the 100 mg CBD in our fed state digestion (22.67 ± 2.1%) compared to 10 mg CBD (14.15 ± 0.6%) (Table 2 and Table S5). The reason might be due to the higher levels of free fatty acids available for use in the 100 mg CBD in in vitro fed digestion, which solubilized the CBD in the AQ fraction in the mixed micelles. We conclude that the test meal containing fat (oil and baby food) and a higher amount of CBD (100 mg) within the diet increases the bioavailability of CBD. Finally, it is advisable to consume CBD with or following a meal containing high fat content to increase bioaccessibility for absorption by epithelial intestinal cells.

### 3.4. MS/MS Survey of Metabolites

**Figure 3:**
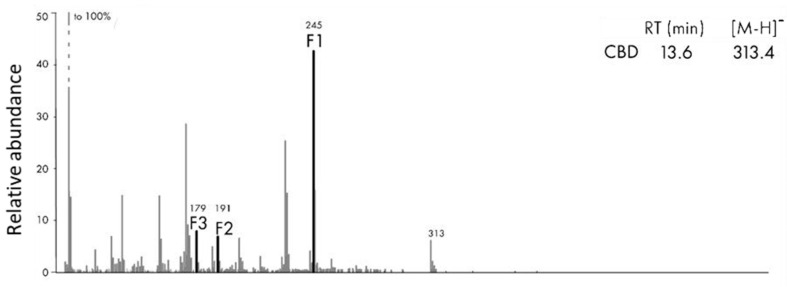
Fragmentation spectrum of AQ fraction of CBD with its corresponding retention time and accurate masses in negative ionization mode that shows the ion fragments using the MS/MS survey. Negative ion mode is observed as deprotonated (H^−^) analyte molecule. [M-H]^−^.

Our MS/MS survey did not identify any known or proposed metabolites of CBD, and only the parent compounds were identified in each phase of digestion using Metabolynx. Only recently have researchers begun to characterize fragmentation patterns of different cannabinoid species by LC-MS/MS [46]. To confirm the presence of cannabinoid species and metabolites in the samples, we performed mass fragmentation characterization using the MS/MS scan of DG and AQ (Figure 3). The most abundant fragmentation ion observed in CBD is 245 *m*/*z*, and several less abundant fragmentation ions (179, 191, 313 *m*/*z*). No metabolites of CBD were observed, including 7-hydroxycannabidiol, and carboxycannabidiol. In Figure 3, representative MS/MS data for CBD collected in negative ion mode are shown.

## 4. Conclusions

In this study we first measured the role of lipase, pancreatin, and bile salts on fatty acid hydrolysis in in vitro digestion. The lipase activity assay results showed an improvement in the fatty acid absorption and lipolysis in the presence of bile. In summary, the lipase enzyme in the presence of pancreatin and bile salt was not inhibited in the absence of supplemental colipase.

In conclusion, fat supplementation of the fed digestion significantly increased the bioaccessibility of CBD. We have shown that a food matrix high in fat affects CBD bioaccessibility and improves the micellarizartion efficiency and recovery of hydrophobic CBD in the AQ fraction. This further supports our hypothesis and suggests that a pre-fed stomach helps increase CBD absorption by the epithelial cells. Finally, it is recommended to consume CBD with food high in fat to increase micellarization of CBD for optimal absorption through the intestinal epithelial cells. Additional work can be done to evaluate metabolism, distribution, and elimination of CBD. For example, in vitro work can be done following artificial digestion using Caco-2 cells and in vivo work using radiolabels to identify and track novel metabolites.

This work fulfills a knowledge gap surrounding the underlying mechanism(s) that explain low oral bioavailability and absorption of CBD. The insight from this work opens doors to future studies in drug formulation and delivery with the goal of improving drug delivery/absorption for improved patient outcomes, i.e., increased onset time to reduce seizure time/severity.

## Figures and Tables

**Table 1:** The % mole conversion of pNPP to NP by lipase and pancreatin activity with and without bile.

Substrate	Assay Components (% Mole Conversion)	
	1:2 lipase:pancreatin	1:2:3 lipase:pancreatin:bile
0.1 mM pNPP	93.6 ± 0.3%	95.5 ± 7.6%
1 mM pNPP	20.6 ± 0.07%	58.6 ± 5.7%

**Table 2:** Cannabidiol percent recoveries from digesta (DG), pellet (P), aqueous (AQ) samples, cannabidiol digestive stability percentages, and cannabidiol bioaccessible micellar efficiencies for fasted and fed state digestions with standard errors (±SE) for 10 mg and 100 mg CBD. Three digestion replicates were used for each fraction.

Samples	Amount of Metabolite in Fasted Digestion		Amount of Metabolite in Fed State Digestion	
	10 mg CBD	100 mg CBD	10 mg CBD	100 mg CBD
DG	72.99 ± 6%	95.67 ± 2%	91.39 ± 25%	83.1 ± 25%
P	92.22 ± 10%	74.10 ± 1.3%	90.15 ± 18%	87.94 ± 29%
AQ	0.69 ± 1.5%	0.15 ± 1%	12.93 ± 7%	18.59 ± 10%
	**Digestive Stabilities**			
	72.99 ± 6%	95.67 ± 2%	91.39 ± 25%	83.1 ± 25%
	**Micellarization Efficiencies**			
	0.65 ± 0.7%	0.14 ± 0.1%	14.15 ± 0.6%	22.67 ± 2.1%

## Data Availability

The data presented in this study are available in supplementary material.

## Supplementary Materials

The following are available online, Table S1: In-vitro human digestion reagents and solutions. Figure S1: Chromatograms with the mass spectrum of standards obtained from UPLC- QTOF-MS analysis of standards of CBD at 13.62 retention time and *m*/*z* 313.4076 in negative mode, Table S2: Inclusion list of metabolites for the CBD with the expected metabolites, ANOVA analysis of variance using Tukey’s Studentized Range (HSD) Test with SAS University Edition for comparison of the %mole conversion of pNPP to NP with and without bile experiment. Tables S3 and S4: ANOVA analysis of variance using Tukey’s Studentized Range (HSD) Test with SAS University Edition for comparison of the AQ and DG phases of digestion in both Fed and fasted state digestion experiments, Table S5: ANOVA analysis of variance using Tukey’s Studentized Range (HSD) Test with SAS University Edition for comparison of micellarization efficiencies of digestion in both Fed and Fasted digestion experiments.

## Sample Availability

Samples of the compounds are not available from the authors.
