# Supplementary material for: The Effects of Food on Cannabidiol Bioaccessibility
Source: Molecules. 2021 Jun 11;26(12):3573. doi: 10.3390/molecules26123573 (PMC8230802; doi:10.3390/molecules26123573)
Supplement: Supplementary file 1 [file molecules-26-03573-s001.zip › molecules-1233163-supplementary.pdf]

Supplementary data

Table S1. *In-vitro* human digestion reagents and solutions

**Gastric and Small Intestinal Phase Solutions**	Pepsin Solution (2mL per reaction)<br>10 mg/mL Pepsin in 0.1M HCl<br>Pancreatin-Lipase Solution (2mL per reaction)<br>20 mg/mL Pancreatin (in 100mM $NaHCO_3$)<br>10 mg/mL Lipase (in 100mM $NaHCO_3$)<br>1.5 mg/mL Colipase (in 100mM $NaHCO_3$)<br>Bile Solution (3mL per reaction)<br>30 mg/mL Bile Extract (in 100mM $NaHCO_3$)
**Preparation of Oral Phase**	Base Solution (q.s. to 1 L with Dl water):<br>Potassium Chloride 1.792g<br>Sodium Phosphate 1.776g<br>Sodium Sulfate 1.140g<br>Sodium Chloride 0.596g<br>Sodium Bicarbonate 3.388g
**For ~10 g Porridge/100mL Oral Phase Solution**	1. Add 100 mL base solution to beaker with stir bar<br>2. Add 40 mg urea<br>3. Add 3 mg uric acid<br>4. Add 5 mg mucin per mL base solution – Note: For 100mL base solution add 500mg.<br>5. Add 3.18 g α-amylase<br>6. Add other enzymes mentioned below right before digestion<br>7. Mix well (at least 15 minutes)
**Enzymes for 100mL Base Solution**	α-amylase –Sigma, A3176. The activity is 15.8 units/mg of solid at pH 6.9 of food to be digested<br>(15.8 units/mg) x (31.8 mg/ml) x (6ml) = 3015 units per digestion<br>ß-glucosidase –0.05mg<br>ß-galactosidase – 0.0034 mg<br>ß-glucuronidase – 0.0008 mg<br>Sulfatase – 0.0065 mg<br>Phosphatase alkaline – 0.25 mg<br>Phosphatase acid – 0.185 mg<br>Neuraminidase – 0.0000024 mg<br>Lysozyme – 0.108 mg

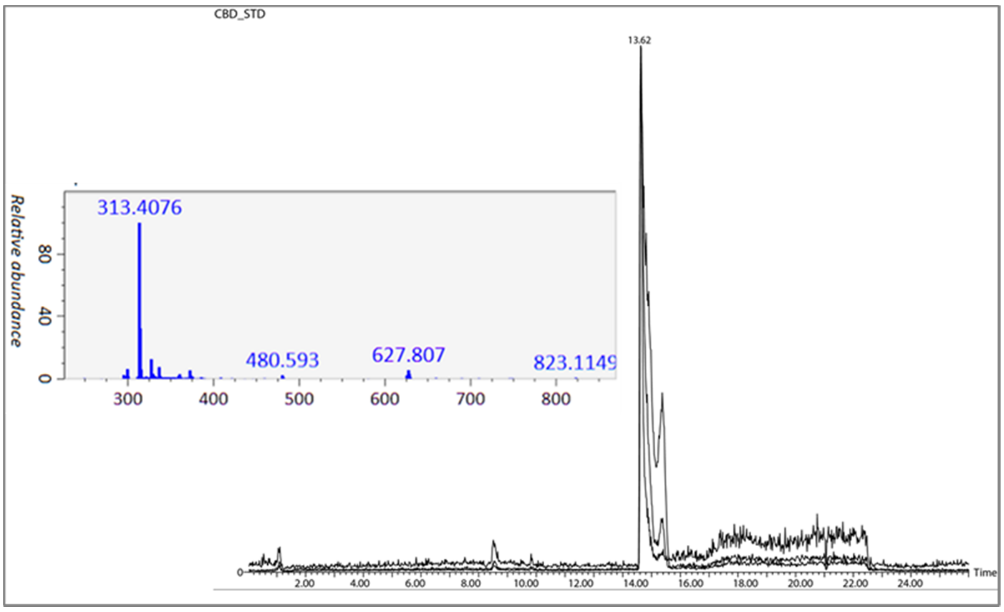

Figure S1. Chromatograms with the mass spectrum of standards obtained from UPLC- QTOF-MS analysis of standards of CBD at 13.62 retention time and m/z 313.4076 in negative mode. The chromatograms were analyzed using the MS-DIAL ver. 4.18. $[M-H]^-$

Table S2. Inclusion list of metabolites for the CBD with the expected metabolites.

Masses	Names
314.4605	CBD
330.4611	7-OH CBD
344.4446	7-COOH CBD

Table S3. ANOVA analysis of variance using Tukey's Studentized Range (HSD) Test with SAS University Edition for comparison of the %mole conversion of pNPP to NP with and without bile experiment.

Comparisons significant at the 0.05 level are indicated by ***.				
Group Comparison	Difference Between Means	Simultaneous 95% Confidence Limits		
0.1 mM pNPP – 1mM pNPP	0.44979	−0.17364	1.07323	
1 mM pNPP – 0.1 mM pNPP	−0.44979	−1.07323	0.17364	

Table S4. ANOVA analysis of variance using Tukey's Studentized Range (HSD) Test with SAS University Edition for comparison of the AQ and DG phases of digestion in both fed and starved digestion experiments.

Tukey's Studentized Range (HSD) Test for Fasted Digestion Percent Recoveries

Comparisons significant at the 0.05 level are indicated by ***.				
Group Comparison	Difference Between Means	Simultaneous 95% Confidence Limits		
DG100mg - DG10mg	18.288	58.658	24.365	***
DG100mg - AQ10mg	92.890	79.561	106.219	***
DG100mg - AQ100mg	93.475	80.146	106.804	***
DG10mg - DG100mg	−18.288	−31.617	−4.959	***
DG10mg - AQ10mg	74.602	61.273	87.931	***
DG10mg - AQ100mg	75.187	61.857	88.516	***
AQ10mg - DG100mg	−92.890	−106.219	−79.561	***
AQ10mg - DG10mg	−74.602	−87.931	−61.273	***
AQ10mg - AQ100mg	0.585	−12.744	13.914	
AQ100mg - DG100mg	−93.475	−106.804	−80.146	***
AQ100mg - DG10mg	−75.187	−88.516	−61.857	***
AQ100mg - AQ10mg	−0.585	−13.914	12.744	

Tukey's Studentized Range (HSD) Test for Fed Digestion Percent Recoveries

Comparisons significant at the 0.05 level are indicated by ***.

Group Comparison	Difference Between Means	Simultaneous 95% Confidence Limits		
DG10mg - DG100mg	3.109	−17.406	23.624	
DG10mg - AQ100mg	67.605	47.090	88.120	***
DG10mg - AQ10mg	74.052	53.537	94.567	***
DG100mg - DG10mg	−3.109	−23.624	17.406	
DG100mg - AQ100mg	64.496	43.981	85.011	***
DG100mg - AQ10mg	70.942	50.427	91.457	***
AQ100mg - DG10mg	−67.605	−88.120	−47.090	***
AQ100mg - DG100mg	−64.496	−85.011	−43.981	***
AQ100mg - AQ10mg	6.447	−14.068	26.962	
AQ10mg - DG10mg	−74.052	−94.567	−53.537	***
AQ10mg - DG100mg	−70.942	−91.457	−50.427	***
AQ10mg - AQ100mg	−6.447	−26.962	14.068	

Tukey's Studentized Range (HSD) Test for Fasted vs Fed Digestion Percent Recoveries

Comparisons significant at the 0.05 level are indicated by ***.				
Group Comparison	Difference Between Means	Simultaneous 95% Confidence Limits		
DG10mg Fasted- DG100mg Fed	3.109	−17.406	23.624	
DG10mg Fasted- DG10mg Fed	67.605	47.090	88.120	
DG100mg Fasted- DG10mg Fed	74.052	53.537	94.567	
DG100mg Fasted - DG100mg Fed	−3.109	−23.624	17.406	
AQ10mg Fasted- AQ100mg Fed	−67.605	-88.120	−47.090	
AQ10mg Fasted- AQ10mg Fed	−64.496	−85.011	−43.981	
AQ100mg Fasted- AQ10mg Fed	6.447	−14.068	26.962	
AQ100mg Fasted - AQ100mg Fed	−74.052	−94.567	−53.537	

Table S5. *ANOVA analysis of variance using Tukey's Studentized Range (HSD) Test with SAS University Edition for comparison of micellarization efficiencies of digestion in both Fed and Starved digestion experiments. ME: Micellarization Efficiency.*

Tukey's Studentized Range (HSD) Test for Fasted Digestion Micellization Efficiencie

Comparisons significant at the 0.05 level are indicated by ***.				
ME Comparison	Difference Between Means	Simultaneous 95% Confidence Limits		
CBD100mg - CBD10mg	0.309	−4.685	5.303	
CBD10mg - CBD100mg	−0.309	−5.303	4.685	

Tukey's Studentized Range (HSD) Test fo Fed Digestion Micellization Efficiencies

Comparisons significant at the 0.05 level are indicated by ***.

ME Comparison	Difference Between Means	Simultaneous 95% Confidence Limits		
CBD100mg - CBD10mg	8.408	2.302	14.514	***
CBD10mg - CBD100mg	−8.408	14.514	−2.302	***

Tukey's Studentized Range (HSD) Test for Fasted vs Fed State Digestion Micellarization Efficiencies

Comparisons significant at the 0.05 level are indicated by ***.

ME Comparison	Difference Between Means	Simultaneous 95% Confidence Limits		
CBD100mg Fasted - CBD10mg Fed	10.447	−11.078	36.872	
CBD10mg Fasted- CBD100mg Fed	89.575	60.914	95.570	***
CBD100mg Fasted– CBD 100mg Fed	91.405	56.731	101.976	***
CBD10mg Fed– CBD10mg Fasted	−25.842	−90.587	−57.701	

Tukey's Studentized Range (HSD) Test for Fasted vs Fed Digestion Digestive Stability

Comparisons significant at the 0.05 level are indicated by ***.				
Digestive Stability Comparison	Difference Between Means	Simultaneous 95% Confidence Limits		
CBD100mg Fasted - CBD10mg Fed	3.127	−13.780	19.487	
CBD10mg Fasted- CBD100mg Fed	−3.109	−19.908	13.764	
CBD100mg Fasted– CBD 100mg Fed	13.845	−6.768	41.314	
CBD10mg Fed– CBD10mg Fasted	−12.602	−24.557	5.598	
